# Lymphocytic Choriomeningitis Virus Meningitis, New York, NY, USA, 2009

**DOI:** 10.3201/eid1602.091347

**Published:** 2010-02

**Authors:** Deborah S. Asnis, Owen Muana, Do Gyun Kim, Minerva Garcia, Pierre E. Rollin, Sally Slavinski

**Affiliations:** Flushing Hospital Medical Center, Flushing, New York, New York, USA (D.S. Asnis, O. Muana, D.G. Kim, M. Garcia); Centers for Disease Control and Prevention, Atlanta, Georgia, USA (P.E. Rollin); New York City Department of Health and Mental Hygiene – Communicable Disease, New York (S. Slavinski)

**Keywords:** Lymphocytic choriomeningitis virus, viral meningitis, aseptic meningitis, arenavirus infections, RNA virus infections, New York City, viruses, dispatch

## Abstract

We describe a case of lymphocytic choriomeningitis virus (LCMV) meningitis in a New York, NY, resident who had no apparent risk factors. Clues leading to the diagnosis included aseptic meningitis during winter and the finding of hypoglycorrachia and lymphocytosis in the cerebrospinal fluid. LCMV continues to be an underdiagnosed zoonotic disease.

Lymphocytic choriomeningitis virus (LCMV) belongs to the family *Arenavirus* and has been found in Europe, the Americas, Australia, and Japan. Arenaviruses are typically associated with rodents, which can become chronically infected. The viruses are zoonotic and transmissible to man. LCMV is primarily maintained in the common house mouse (*Mus musculus* and *M. domesticus*), but has also been reported among pet and research rodents, including hamsters and guinea pigs (*1*). The prevalence of LCMV in wild mice in the United States ranges between 3.9% and 13.4% (*2*). Human cases demonstrate a fall-to-winter predominance corresponding to the movement of rodents indoors (*1*). Limited data are available on human infections but serologic studies in Washington, DC, Baltimore, Maryland, and Birmingham, Alabama, identified evidence of previous infections among 2%–10% of the population sampled (*3*). A study in a Washington, DC, military base found that 8%–11% of patients having a febrile neurologic illness between 1941 and 1958 were seropositive for LCMV(*4*)

Transmission occurs by inhalation (aerosol and droplet), fomites, or direct contact with excreta or blood from infected rodents. Human-to-human transmission has been reported during pregnancy from infected mothers to the fetus (*5*) and through solid organ transplantation (*6*). The incubation period is 1–2 weeks. Infections in persons with an intact immune system are often asymptomatic or result in a mild self-limiting illness including fever, chills, myalgia, and headache. Photophobia, anorexia, testicular or parotid pain, pharyngitis, and cough have also been noted (*1*). Leukopenia, thrombocytopenia, and mild liver function abnormalities are common and may last 1–3 weeks. Central nervous system invasion is seen only in a few patients either after an initial febrile illness or, less commonly, without any early symptoms. During this neurologic phase, most patients have aseptic meningitis and a peripheral leukocytosis (*1*). Cerebrospinal fluid (CSF) leukocyte cell counts often exceed 1,000/μL, and glucose levels are low. Infection is rarely fatal, although ascending paralysis, transverse myelitis, or encephalitis may develop (*1*).

Critical or even fatal outcomes from LCMV are usually associated with transplacental infections, and, more recently, solid organ transplantation. Congenital LCMV can result in hydrocephalus, chorioretinopathy, macrocephalopathy, or microcephalopathy and can mimic the classic TORCH (toxoplasmosis/*Toxoplasma gondii*, other infections, rubella, cytomegalovirus, herpes simplex virus) pathogens. In 1 study of 26 infants with congenital LCMV, 9/26 (35%) died and 10/16 (63%) survivors had severe neurologic sequelae (*7*). Transplacental infection presumably occurs after maternal viremia during the first and second trimesters (*8*). In 2003 and 2005, the first reports of infection after solid organ transplantation were reported in which 7 of 8 patients receiving LCMV infected organs, kidney, liver, and lung, died (*6*). Therapy for LCMV is supportive, although limited data support the use of ribavirin in immunosuppressed patients. We report a case of meningitis caused by LCMV in New York City.

## The Patient

A 49-year-old man (taxi driver) sought treatment during the winter of 2009. He had a 7-day history of fever, chills, headache, nausea, vomiting, neck rigidity, generalized weakness, and paresthesias in his fingers. He denied recent travel or exposure to sick contacts. Medical history included a corneal transplant the previous year. His temperature when admitted was 37.5°C. Physical examination identified nuchal rigidity but was otherwise unremarkable. CSF showed leukocytes 871 cells/μL; erythrocytes 2 cells/μL, with 93% lymphocytes and 7% monocytes; glucose 32 mg/dL (serum glucose 93 mg/dL); and protein 185 mg/dL. Results of a computerized axial tomography scan of the brain and chest radiograph were unremarkable. A complete blood count and results of serum chemical tests and liver function tests were unremarkable. Blood cultures yielded no growth. CSF cultures for bacterial, fungal, and mycobacterial organisms were negative. Results for syphilis rapid plasma reagin, cryptococcal antigen, and HIV testing were negative.

Because of the patient’s hypoglycorrachia, pronounced CSF leukocytosis, and negative CSF cultures, acute infection with LCMV was considered. Serum tested at a commercial laboratory was positive for LCMV (immunoglobulin [Ig] G titer >256 (reference <16) and IgM titer 80 (reference <20) by immunofluorescence assay. CSF collected on day 1 and serum collected on days 7 and 15 was tested for LCM antibodies by the Centers for Disease Control and Prevention’s Special Pathogens Branch using ELISA. CSF and serum were both IgM and IgG positive and a 4-fold rise in acute-phase and convalescent-phase serum titers indicated the patient had a recent infection with LCMV (Table). The patient improved with supportive care, recovered fully, and was discharged on day 12.

**Table:** CSF and serum LCM virus ELISA titers for specimens obtained from a male patient, New York, NY, USA, 2009*

Hospitalization day	Specimen type	LCM IgM ELISA	LCM IgG ELISA
1	CSF	1,280	QNS
7	Serum	6,400	100
15	Serum	6,400	400

*CSF, cerebrospinal fluid; LCM, lymphocytic choriomeningitis; Ig, immunoglobulin; QNS, quantity not sufficient. Titers <100 in serum and <25 in CSF are considered negative.

## Conclusions

The incidence rate of LCMV infection in New York City is unknown. The disease is likely underdiagnosed because only a limited number of commercial laboratories offer LCMV testing. Diagnosed cases of LCMV are often identified because they are part of a larger outbreak. Since 1960, only 7 cases have been reported in the literature (*8**,**9*); all but 2 had had contact with rodents. Several large outbreaks have been reported, often associated with pet hamsters or laboratory mice or hamsters; 1 outbreak had >236 human cases (*8*). A total of 54 cases of congenital LCMV are cited in the literature, 34 since 1993 (*5*).

Our patient denied both hamster and mouse exposure. His corneal transplant had occurred >1 year before, and was unlikely to be the source of infection. A definitive diagnosis was difficult, but testing by Centers for Disease Control and Prevention confirmed the diagnosis.

New York City Health Department officials attempted an investigation of the patient’s home and surroundings to look for evidence of rodents or rodent excreta, but were unable to do so due to the patient’s noncompliance. Several clues have assisted clinicians in the identification of patients with acute LCMV infection: 1) aseptic meningitis or encephalitis during the fall–winter season, 2) a febrile illness followed by brief remission before onset of neurologic illness, and 3) CSF with a lymphocytosis and hypoglycorrachia. Patients with suspected LCMV should be asked about potential rodent exposure (*1*). Patients with a history of organ transplantation within the preceding 3 months should be fully evaluated to determine whether infected organs were the source of LCMV.

We recommend enzyme immunoassay-based testing of CSF and serum when LCMV is considered. Public health surveillance, rodent control, healthcare provider education, and improved laboratory testing can enhance recognition of illness.

Health departments should consider making LCMV a reportable condition, and, if aseptic meningitis is reportable, questions should be added to case investigation forms regarding rodent exposure. LCMV was made reportable in New York City in 2009. Only with a better understanding of the true incidence of LCMV will authorities be able to enact measures to better prevent and control this disease in the vulnerable sections of our society.
